# Description of a New Simple and Cost-Effective Molecular Testing That Could Simplify *MUC1* Variant Detection

**DOI:** 10.1016/j.ekir.2024.01.058

**Published:** 2024-02-03

**Authors:** Victor Fages, Florentin Bourre, Romain Larrue, Andrea Wenzel, Jean-Baptiste Gibier, Fabrice Bonte, Claire-Marie Dhaenens, Kendrah Kidd, Stanislav Kmoch, Anthony Bleyer, François Glowacki, Olivier Grunewald

**Affiliations:** 1 Nephrology, Centre Hospitalier Regional Universitaire de Lille, Lille, France; 2 Service de Toxicologie et Génopathies, CHU Lille, Lille, France; 3 Institute of Human Genetics, Center for Molecular Medicine Cologne, Cologne, Germany; 4 Jean-Pierre Aubert Research Center, University of Lille, Lille, France; 5 Functional and Structural Platform, Université de Lille, Lille, France; 6 Department of Biochemistry and Molecular Biology, Institut National de la Santé et de la Recherche Médicale, Centre Hospitalier Universitaire de Lille, Lille, France; 7 Section on Nephrology, Wake Forest School of Medicine, Winston-Salem, North Carolina, USA; 8 First Faculty of Medicine, Charles University, Nové Město, Czechia; 9 Neuroscience and Cognition, University Lille, Inserm, CHU Lille, Lille, France

**Keywords:** ADTKD, *MUC1*, VNTR

## Abstract

**Introduction:**

Patients with autosomal dominant tubulointerstitial kidney disease (ADTKD) usually present with nonspecific progressive chronic kidney disease (CKD) with mild to negative proteinuria and a family history. ADTKD-*MUC1* leads to the formation of a frameshift protein that accumulates in the cytoplasm, leading to tubulointerstitial damage. ADTKD-*MUC1* prevalence remains unclear because *MUC1* variants are not routinely detected by standard next-generation sequencing (NGS) techniques.

**Methods:**

We developed a bioinformatic counting script that can detect specific genetic sequences and count the number of occurrences. We used DNA samples from 27 patients for validation, 11 of them were patients from the Lille University Hospital in France and 16 were from the Wake Forest Hospital, NC. All patients from Lille were tested with an NGS gene panel with our script and all patients from Wake Forest Hospital were tested with the snapshot reference technique. Between January 2018 and February 2023, we collected data on all patients diagnosed with *MUC1* variants with this script.

**Results:**

A total of 27 samples were tested anonymously by the BROAD Institute reference technique for confirmation and we were able to get a 100% concordance for MUC1 diagnosis. Clinico-biologic characteristics in our cohort were similar to those previously described in ADTKD-*MUC1.*

**Conclusion:**

We describe a new simple and cost-effective method for molecular testing of ADTKD-*MUC1.* Genetic analyses in our cohort suggest that *MUC1* might be the first cause of ADTKD. Increasing the availability of *MUC1* diagnosis tools will contribute to a better understanding of the disease and to the development of specific treatments.

## Graphical abstract

**Figure:**
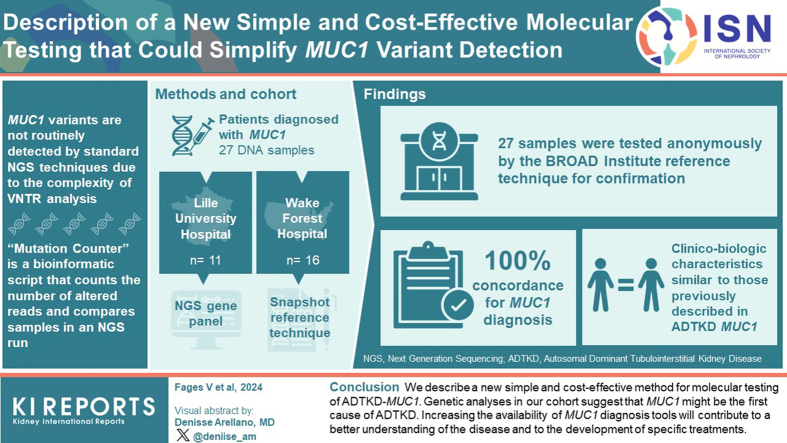

ADTKD is a group of hereditary kidney diseases characterized by tubular damage and interstitial fibrosis of the kidney leading to end-stage renal disease (ESRD).^1^ Patients with ADTKD usually present with nonspecific progressive CKD with mild to negative proteinuria and a family history.^2^ Several genes have been associated with ADTKD, notably *UMOD*, encoding uromodulin; *MUC1*, encoding transmembrane epithelial mucin 1; *REN*; *HNF1B*; and *SEC61A1*.^3^ According to the latest studies, *UMOD* represents the most frequent subtype of ADTKD with estimated prevalence of 37.1% followed by *MUC1* in 35% of families that are *UMOD*-negative with an estimated overall prevalence of 21%.^4^

ADTKD is presumed to be underdiagnosed, given the nonspecificity of the phenotypic characteristics. ADTKD-*MUC1* pathogenic variations of *MUC1* are located in a repetitive sequence of 60 base pairs in the variable number tandem repeat (VNTR) domain of the gene. This sequence variant leads to the formation of an MUC1 frameshift protein that accumulates in the cytoplasm, leading to abnormal intracellular transport and tubulointerstitial damage.^4^, ^5^, ^6^ VNTR are genomic sequences 7 to thousands of base pairs long that are repeated in tandem from 2 to hundreds of times.^4^ In humans, VNTRs have been implicated in monogenic disorders such as *MUC1*; however, VNTR polymorphisms also seem to influence several kidney disease related characteristics.^4^ VNTRs also have consequences beyond kidney diseases because they are known to have a functional role in genome regulation and as biomarkers, notably in neurology and oncology, and their analysis remains challenging.^7^^,^^8^

*MUC1* VNTR is particularly difficult to assay because it consists of many copies of a large repeat unit (60 base pairs) with a very high G-C content (>80%).^5^ Thus, ADTKD-*MUC1* prevalence remains unclear because *MUC1* variants are not routinely detected by standard NGS techniques due to difficulties in interpreting and reassembling sequences in areas of repetitive DNA. Consequently, ADTKD-*MUC1* is probably underdiagnosed and require specialized genetic testing that are not yet clinically available.^1^^,^^5^

We describe a new simple and cost-effective method for molecular testing of ADTKD-*MUC1* and we describe ADTKD-*MUC1* clinical and genetic characteristics in our cohort.

## Methods

### Participants

Between January 2018 and February 2023, we collected data on all patients diagnosed with *MUC1* variants in Lille University Hospital. Genetic analysis were sent from different hospitals across France. Clinico-biologic characteristics were recorded, including longitudinal estimated glomerular filtration rate and renal outcomes. Participants with suspected ADTKD according to their referral nephrologist had a positive family history compatible with autosomal dominant inheritance of CKD with features of ADTKD, including progressive loss of kidney function, bland urinary sediment, absent to mild albuminuria, normal sized or small sized kidneys. Minor patients were excluded from the study. Genetic analyses were performed after obtaining written consent from patients.

### Ethical Statement

This study was performed according to the Declaration of Helsinki and the Declaration of Istanbul. Ethical committee was bypassed according to French laws and the local institutional review board (Centre Hospitalier Universitaire de Lille) because the study was retrospective and observational. Informed consent was obtained from all subjects. Patients and laboratory data were analyzed anonymously and registered with the French data protection registry (Commission Nationale de l’Informatique et des Libertes, i.e., CNIL), referenced #DEC22-308.

### DNA Analysis – Genetic Testing

#### Panel NGS 107 – Haloplex, MUC1

For this work, we used an NGS gene panel of 107 genes known to be involved in inherited kidney disease. This panel contains all 5 ADTKD described genes, *MUC1* being one of those genes. This panel has been used in routine molecular diagnosis of CKD in our molecular biology department in the Lille University Hospital in France since 2017. We used the library preparation kit Haloplex of the society Agilent. Reference of the kit is ID 22339-1591352935. Libraries were sequenced with a Miseq or a NextSeq550 Illumina sequencer. This technique provides large depth of coverage of the *MUC1* VNTR, over 1000x for each one of the sequenced samples. We used a minimum of 16 samples per run.

### Bioinformatic Script, Description, and Validation

#### Description of the Script Mutation Counter

In order to detect very low rate of altered reads, we used a bioinformatic counting script (Bioinformatic counting R script [Mutation Counter]), called Mutation Counter, developed by the bioinformatic team of the Lille University Hospital. This script can detect specific genetic sequences in the sequencing data and count the number of occurrences. We input in the settings all 5 described VNTR *MUC1* variations,^4^ the wild type sequence and a specific “All” sequence searching for any undescribed variation of the 7 cytosine homopolymers:

• MUT_27dupC : GGGCTCCACCGCCCCCCC**C**AGCCCACGGTGTC
• MUT_27insCCCC : GGGCTCCACCGCCCCCCC**CCCC**AGCCCACGGTGTC
• MUT_26_27insG: GGGCTCCACCGCCCCCC**G**CAGCCCACGGTGTC
• MUT_28dupA: GGGCTCCACCGCCCCCCCA**A**GCCCACGGTGT
• MUT_23delinsAT : GGCTCCACCGCC**AT**CCCCAGCCCACGGTGTC
• WT : GGGCTCCACCGCCCCCCCAGCCCACGGTGTC
• All : GGGCTCCACCG.**∗**CCCCCCCAGCCCACGGTGTC

#### Bioinformatic Analysis

For each variation, a Fischer statistic test was applied to compare samples in an NGS run. A *P*-value < 0.001 was considered as significative, meaning the sample contains a significantly higher number of reads presenting the variation compared to the other samples in the same run, and was considered as a positive carrier of the variation. Examples of the output of the bioinformatic run are presented in Supplementary Table S1 and Supplementary Figure S1.

#### Validation of the Technique

To test this novel molecular testing, we used DNA samples from 27 patients. Of these, 11 were patients from the Lille University Hospital in France (7 patients with clinically suspected ADTKD and 4 major asymptomatic adult children of an ADTKD-*MUC1* patient). Sixteen were from the Wake Forest Hospital, NC. All participants agreed to specific *MUC1* molecular testing. Samples were anonymized before testing. All 27 samples were tested anonymously by the BROAD Institute reference technique for confirmation. Their technique was considered as the gold standard to detect MUC1 variant.^5^

### Statistical Analysis

Quantitative parameters are presented as median and interquartile range or mean and SD when appropriate, and qualitative parameters are presented as fractions with percentages. Kaplan-Meier curves were generated to display ESRD-free survival. Patients who had not reached ESRD at the end of the study (outcome of interest not occurred during follow-up time) were considered censored individuals. Censoring time was defined as age at last follow-up. Data were analyzed with R software version 3.6.1 (R Foundation for Statistical Computing, Vienna, Austria).

## Results

### Validation of the Novel Molecular Testing Tool for *MUC1* Variant Detection

Eleven patients from the Lille University Hospital in France (7 patients with clinically suspected ADTKD that were positive with our technique and 4 major asymptomatic adult children of an ADTKD-*MUC1* patient who were all negative with our technique) were anonymously sent to the reference center at the Broad Institute. All results were confirmed with the snapshot reference technique. Sixteen patients from the Wake Forest Hospital, NC (8 positive and 8 negative for *MUC1* with the snapshot reference technique at the Broad Institute) were anonymously sent to our department in Lille. With our technique, we were able to get a 100% concordance with results from the gold standard (Supplementary Figure S2).

### Identification of Pathogenic Variants by NGS

Between January 2018 and February 2023, genetic analysis was performed using NGS and our new bioinformatic script in 886 patients with suspected nonglomerular nephropathy. One hundred thirty-seven patients had clinically suspected ADTKD. Variants in the following genes were detected: *MUC1* (*n* = 19), *UMOD* (*n* = 7), *HNF1B* (*n* = 3), *COL4A3* (*n* = 1), *PAX2* (*n* = 3), *NPHP1* (*n* = 1), *NPHP4* (*n* = 1), *TTC21B* (*n* = 1), *CLCN5* (*n* = 1), *REN* (*n* = 1), and *NRIP1* (*n* = 1). Among the 19 patients positive for *MUC1*, 7 were confirmed with the snapshot reference technique, their samples were used for the validation of the technique as described above. Eighteen of 19 patients positive for ATDKD-*MUC1* had a cytosine insertion in the *MUC1* VNTR; 1 had a new sequence variant (21insG). Median delay between first nephrology visit and genetic diagnosis was 6 (interquartile range: 2.2–11) years.

### Clinical Characteristics of Patients With ADTKD-*MUC1*

Clinical characteristics are summarized in Table 1. Twelve of 19 patients were female. The mean age at presentation was 36 years. Nearly all patients had a positive CKD family history. All had proteinuria <0.3 g/d. In 8 patients who underwent renal biopsies, we found nonspecific lesions of secondary glomerulosclerosis and tubulointerstitial kidney disease. Four biopsies revealed microcystic dilatation of the tubules. The estimated glomerular filtration rate at diagnosis was 43 (±19) ml/min per 1.73 m^2^. More than half of the patients had renal cysts, mainly small bilateral cysts. The median age at ESRD was 37.5 (interquartile range: 35–50.5) years. A survival curve to age of ESRD is shown in Supplementary Figure S3. No specific extra-renal manifestation were found.

**Table 1:** Clinical characteristics of patients with ADTKD-*MUC1*

Characteristics	Patients with ADTKD-*MUC1* (*N* = 19)
Sex, female (%)	12/19 (63)
Age at presentation (yr)	36 (28,48)
Proteinuria (g/l)	0.09 (±0.06)
Hypertension (%)	4/16 (25)
Positive CKD family history (%)	16/17 (94)
eGFR at diagnosis (ml/min per 1.73 m^2^)	43 (±19)
ESRD	8/16 (50)
Age at ESRD (years)	37.5 (35;50.5)
Renal cysts (%)	8/15 (53)
Gout (%)	2/17 (12)
Number of families	15

ADTKD, autosomal dominant tubulointerstitial kidney disease; CKD, chronic kidney disease; eGFR, estimated glomerular filtration rate; ESRD, end-stage renal disease (dialysis or preemptive kidney transplant).

Quantitative parameters are presented as mean ± SD or median (interquartile range).

Qualitative parameters are presented as fractions with percentages.

## Discussion

Diagnosis of ATDKD-*MUC1* remains challenging and is probably underdiagnosed due to the lack of availability of clinical genetic testing. The latest Kidney Disease Improving Global Outcomes Consensus Report on ADTKD suggests referral to specialized centers.^1^ In 2021, Bleyer *et al.*^9^ suggested the use of an inherited kidney disease multigene panel, including *UMOD*, *REN*, and other genes of interest, with the use of a laboratory specifically performing *MUC1* targeted analysis if this multigene panel was negative. Indeed, due to the high guanosine-cytosine content and the repetitive nature of VNTR sequences, *MUC1* variants cannot be identified by commonly used sequencing methods (NGS, exome sequencing).^9^ *MUC1* diagnosis can only be done in centers performing targeted analysis for *MUC1* frameshift variants, such as the CLIA-certified Broad Institute of Harvard and Massachusetts Institute of Technology.^9^ We describe for the first time a new simple and cost-effective method that could simplify ADTKD-*MUC1* diagnosis.

We found in our cohort characteristics similar to those previously described in ADTKD-*MUC1* patients. The age of presentation was 36 years, earlier than in the ADTKD-*MUC1* cohort described by Olinger, which was 47 years.^4^ In our study, the age at ESRD was 37.5 (35–50.5) years. In a Japanese cohort of ATDKD-*MUC1* variants, (*n* = 31 with the inclusion of family affected patients), ESRD was described at a later stage with a median age at ESRD of 45years.^10^ Olinger *et al.*,^4^ found a median age at ESRD of 36 years in their ADTKD-*MUC1* cohort, similar to ours. This difference could be explained by the exclusion of family members in our cohort and in Olinger’s. Phenotypes can be variable^11^; in a study of 186 families and 95 patients with *MUC1* variants, Bleyer *et al.*^12^ described a variable age of onset of ESRD within and between families, ranging from 16 to >80 years. Occasional renal cysts can be found; however, their frequency appears to be no higher than in other noncystic kidney diseases. In their study, Bleyer *et al.*^12^ found no medullary cysts on renal ultrasound examination of 35 individuals. In our study, half of patients had renal cysts of variable localization and size. Gout was rare, which was expected with *MUC1* variant. Gout is more prevalent and of earlier onset in ADTKD-*UMOD* compared to *MUC1*.^4^ As described previously, MUC1 frameshift protein accumulates in several tissues^13^ but does not cause extra-renal manifestations. We did not find evidence of specific clinical manifestations besides the kidney in our study. Of note, 1 patient had a lymphangioleiomyomatosis with cystic lung lesions, with no evident link with ADTKD-*MUC1*. *MUC1* is known to be involved in cancer invasion, metastasis, and angiogenesis, through its role in intracellular signaling processes.^14^ As described in the literature, we did not find an increased risk of cancer in our cohort. Histology revealed tubular microcystic in 4 of 8 biopsies. We did not perform noninvasive immunohistochemical method to detect MUC1 frameshift proteins because we did not have access to this technique in our center.

Penetrance is known to be complete and there are no genotype-phenotype correlations. In our cohort, all patients but 1 had positive CKD family history. Individuals with no family history are unlikely to have ADTKD-*MUC1* but it should not completely rule out the diagnosis because *de novo* variants may occur.^9^ All but 1 patient had a cytosine insertion in *MUC1*-VNTR, which is the most frequent sequence variant described in the literature. In Bleyer *et al.*^12^ study, all families were found to have an additional cytosine insertion in the VNTR of *MUC1*, suggesting that the neoprotein created because of this insertion is central to the pathogenesis of the kidney disease. Among the 93 families with ADTKD-*MUC1* in Olinger *et al.*^4^ study, 87 presented with a cytosine duplication (27dupC, 93.5%), 3 with an adenine duplication (28dupA, 3.2%), 2 with a guanine insertion (26_27insG, 2.2%), and 1 with a small indel (23delinsAT, 1.1%). 27dupC is the most commonly reported *MUC1* sequence variant in studies in Europe, United States, and Japan.^4^^,^^10^ In our study, 1 patient had a new variant, 21insG, not previously described in the literature, which leads to a frameshift variation and a truncated MUC1 protein. The consequences on the frameshift protein are probably similar to those with a cytosine insertion. Recently, a novel variant located before the first VNTR repeat unit was identified in a Dutch family.^15^

*MUC1*-ADTKD is probably underdiagnosed because its diagnosis requires specialized genetic testing due to the complexity of VNTR analysis. ADTKD-*UMOD* is considered the most frequent cause of ADTKD.^16^ In the International ADTKD cohort, ADTKD-*UMOD* represented the most frequent subtype of ADTKD with an estimated prevalence of 37.1% followed by ADTKD-*MUC1* in 35.1% of families *UMOD* negative.^4^ However, in a Spanish cohort, *MUC1* variant was the main genetic cause of ADTKD.^11^ Lately, Okada *et al.*^10^ used long-read sequencing to detect *MUC1* variant; ATDKD-*MUC1* was most commonly found with *MUC1* variants in 9 patients, whereas 6 had *UMOD* variants. In their study, diagnostic rate of *MUC1* variants in patients with clinically suspected ADTKD is 18.8%. In our study, ATDKD-*MUC1* also appears to be more frequent than *UMOD*.

Other identified genes with pathogenic variants were *HNF1B* (*n* = 3), *REN* (*n* = 1), *COL4A3* (*n* = 1), *PAX2* (*n* = 3), *NPHP1* (*n* = 1), *NPHP4* (*n* = 1), *TTC21B* (*n* = 1), *CLCN5* (*n* = 1), *NRIP1* (*n* = 1). Patients with HNF1B sequence variants are usually distinguishable because they present with extra-renal manifestations such as diabetes, gout and hyperparathyroidism, hypomagnesemia, elevated liver enzymes, and congenital anomalies of the kidney and urogenital tract.^17^ *HNF1B* prevalence is probably underestimated in this study, because we focused on patients with suspected ADTKD, whereas *HNF1B* diagnosis can also be made with genetic testing for diabetes or congenital anomalies of the kidney and urogenital tract. Patients with *REN* variants also usually have specific characteristics compared to other ADTKD, such as childhood anemia, mild hypotension, and hyperkalemia.^18^ Other variants found are consistent with the literature on ADTKD. Wopperer *et al.*^19^ performed exome sequencing in 16 families with ADTKD with no sequence variant found (including *MUC1*), of which 9 showed diagnostic variants in the nephrome (4 in *COL4A5*, 2 in *INF2*, and 1 each in *COL4A4*, *PAX2*, *SALL1*, and *PKD2*). Nephronophthisis (1 *NPHP1*, 1 *NPHP4*, and 1 *TTC21B* found in our study) may also be a differential diagnosis of ADTKD in young adults; however usually, autosomal recessive inheritance distinguishes it from ADTKD.^1^^,^^20^ *NRIP1* and *CLCN5* are less frequently described in patients with clinical features of ADTKD.

Thousands of different VNTR polymorphisms are known in the human genome, and they have been implicated in monogenic disorders but could also contribute to complex traits, including kidney-related traits.^21^^,^^22^ VNTR length and polymorphisms could also have functional consequences and need to be considered more in research of complex disorders.^23^ Considering that VNTR’s are difficult to analyze, their role in physiology is still not fully understood.^7^ The difficulty in VNTR analysis lies in the detection of a variant within a 60 base pair repeat unit of a VNTR sequence. Indeed, repeated regions in the DNA cause difficulties for the alignment of sequences and genome assembly, making it difficult to discern where the repeat lies, their true copy numbers and whether the repeat is polymorphic.^23^ Considering this, we understand that ADTKD-*MUC1* diagnosis is complex and that *MUC1* pathogenic variants have been identified only recently by Kirby *et al.*^5^ because they lie in *MUC1* VNTR region, which consists of many copies of a large repeat unit (60 base pairs) with a very high G-C content (>80%). As seen previously, *MUC1* variants are not routinely detected by NGS which uses short-read sequencing.

We describe a new simple and cost-effective method for molecular testing of ADTKD-*MUC1* using NGS. This technique uses a script “Mutation Counter” that counts the number of altered reads and compares samples in an NGS run, in order to identify patients with significantly higher number of reads presenting the variation compared to the other samples. All samples anonymously tested, positive and negative, have been confirmed by the Broad Institute reference technique.^9^ This technique could ease ATDKD-*MUC1* diagnosis in clinical practice and unveil a higher prevalence of the disease. The major advantage of our technique is that it does not require any additional technical analysis and it simply relies on NGS data analysis. Therefore, it is possible to test concomitantly *MUC1* and other ADTKD genes. Testing all 5 genes (*MUC1*, *UMOD*, *REN*, *HNF1B*, and *SEC61A1*) at the same time in NGS will be more efficient and at no additional cost. Improving detection of sequence variants within the VNTR might also impact diagnosis of other diseases. For example, variants in carboxyl ester lipase gene VNTR have been associated with diabetes and exocrine pancreatic dysfunction.^24^

This technique has several limitations. First, the counting script only detects determined genetic sequences and cannot detect an unknown variation. We prevented this issue with a generic seed that can detect any undescribed variation of the 7 cytosine homopolymer of the VNTR. Using this technique, we were even able to detect a novel sequence variant, 21insG. We were able to get a 100% concordance with results from the gold standard on the 27 samples that were tested with both techniques. However, we cannot exclude false negatives. If the *P*-value is equivocal, the best diagnostic strategy would be to send the sample to a specialized center to test the patient with the snapshot reference technique. Second, the counting script could not work if all 16 samples in the run were *MUC1*-positive. However, with our NGS panel 107, we analyzed samples of patients with inherited kidney disease from various origins and it seems very unlikely to only have samples from *MUC1* patients within the same run. If a doubt persists for a patient, he could be tested in another run with different samples or even with specific “control” samples from patients with no renal disease history. Third, this technique does not identify precise positioning of sequence variant in the VNTR. This position is not correlated to the phenotype but could be relevant for familial segregation studies. The best technique to determine precise positioning of the causative variant and to identify sequence variants in ATKD-*MUC1* is long read single molecule real time sequencing.^25^ This technique is able to identify potential novel sequence variants occurring within the VNTR, contrary to the snapshot reference technique that can only detect cytosine insertion variant. Single molecule real time sequencing for *MUC1* is still expensive and is only done in few specialized centers. Lastly, in order to detect a small number of sequences with variant, we need a high depth of coverage. The Haloplex kit of Agilent allows a depth of 1000x, but other NGS Library Preparation Kits may not have this resolution. This technique could also be difficult with whole-exome sequencing or whole-genome sequencing with depths usually around 50x to 100x. Recently, a French team developed a diagnostic algorithm with a computational pipeline that can be used on short read sequencing data.^26^ Further studies are needed to assess its use with whole-genome sequencing or whole-exome sequencing and to determine the optimal depth to identify true positives.

Currently, *MUC1* diagnosis cannot be done routinely in clinical laboratories; however, the increasing availability of long read sequencing methods should improve this diagnosis and lead to a greater understanding of underlying physiology and disease. Noninvasive immunohistochemical method could also be useful in the future for diagnostic testing but are not yet available in clinical practice.^13^ Improving *MUC1* variant detection with routine techniques seems essential, especially because targeted therapies are being studied but no specific treatment has been validated yet. Dvela-Levitt *et al.*^27^ reported that a small molecule BRD4780 reroutes *MUC1* for lysosomal degradation and could be a promising lead for the treatment of toxic proteinopathies.

In conclusion, we describe a novel simple technique to detect *MUC1* variant with a bioinformatic script using NGS panel sequencing. Genetic analyses in our cohort suggest that *MUC1* might be the first cause of ADTKD. Increasing the availability of *MUC1* diagnosis tools will contribute to a better understanding of the disease and to the development of specific treatments.

## Disclosure

All the authors declared no competing interests.

## Supplementary Material

Supplementary File (PDF)

Bioinformatic counting R script (Mutation Counter).

**Figure S1.** Graphical representation of the percentage of reads expressing the mutation 27dupC.

**Figure S2.** Validation of the Mutation Counter script.

**Figure S3.** Freedom from end-stage kidney disease (ESKD).

Kaplan Meier curve of renal survival in patients with ADTKD-MUC1.

**Table S1.** Example of the output of the bioinformatic run “Mutation Counter.”
