# Supplementary material for: Description of a New Simple and Cost-Effective Molecular Testing That Could Simplify MUC1 Variant Detection
Source: Kidney Int Rep. 2024 Feb 3;9(5):1451–7. doi: 10.1016/j.ekir.2024.01.058 (PMC11068942; doi:10.1016/j.ekir.2024.01.058)
Supplement: Supplementary File (PDF) [file mmc1.pdf]

Bio-informatic counting R script (Mutation Counter)

```
#!/usr/bin/env Rscript
version=0.1
## Author: Martin Figeac
## usage : Rscript do_it.R design.txt CTL MUT {MUT}
##
## usage : Rscript do_it.R count.txt ALL delC dupC

##
## Compute a few stats on all count data
## Warning : will substract MUT from CTL (CTL must be 'ALL')

## at least one count for each sample muste be over this threshold
MIN_COUNTS=100

cat ("## script do_it.R running in version", version,"\n");
cat ("## date is", date (), "\n");
args=commandArgs(TRUE)
cat ("opening file", args[1], "...");
csv=read.csv (args[1], sep=" ", header=TRUE, as.is=TRUE, comment.char="#");
cat ("done\n");

if (args[2] %in% colnames(csv)) {
  ctl=which (args[2] == colnames(csv));
} else {
  cat ("Erreur argument for control : ", args[2], " not found in count
 file", args[1], "\n");
  cat ("colnames = ", colnames(csv),"\n");
  exit (1)
}

if (length(args) < 3) {
  cat ("Erreur usage, need at least 3 arguments : Rscript do_it.R count.txt
 CTL MUT1 {MUT2}\n");
  exit (1);
}

for (mutationsArg in 3:length(args)) {
  if (args[mutationsArg] %in% colnames(csv)) {
    mut=which (args[mutationsArg] == colnames(csv));
  } else {
    cat ("Erreur argument for mutation : ", args[mutationsArg], " not found
 in count file", args[1], "\n");
    cat ("colnames = ", colnames(csv),"\n");
    exit (1)
  }
  cat ("\n##\n");
  cat ("## Testing mutation", args[mutationsArg], "against control",
 args[2],"\n");

  rows=c()
```

```
  for (sample in 1:nrow(csv)) {
    if (any (csv[sample,c(mut, ctl)] >= MIN_COUNTS)) {
      rows=c(rows, sample);
    } else {
      cat ("WARNING : Sample ", csv[sample,1], " doesn't have enough counts
(", MIN_COUNTS, ") and has been excluded\n", sep="");
    }
  }

  cat ("\n");
  cat ("Fisher Matrice :\n");
  pMatrix = matrix(0,nrow=length(rows), ncol=length(rows));
  colnames(pMatrix)=rownames(pMatrix)=substr(csv[rows, 1], 1, 20);
  i=j=1;

  cat ("Samples", substr(csv[rows,1], 1,20), sep="\t"); cat ("\n");
  for (sample1 in rows) {
    cat (substr(csv[sample1, 1], 1,20), "\t", sep="");
    for (sample2 in rows) {
      m=matrix (c(csv[sample2, ctl]-csv[sample2, mut], csv[sample1, ctl]-
csv[sample1, mut], csv[sample2, mut],csv[sample1, mut]), nrow=2);
      p=fisher.test (m, alternative="gr")$p.value;
                                         #    p=fisher.test (m,
alternative="t")$p.value;
                                         #    p=chisq.test (m)$p.value;
      pMatrix[i,j]=p;
      cat (p, "\t", sep="");
      j=j+1;
    }
    cat ("\n");
    i=i+1;
    j=1;
  }

  cat ("\n");

  for (sample in 1:nrow(pMatrix)) {
    pMatrix[sample,sample] = NA;
  }

  for (pos in 1:min(6,nrow(pMatrix)-1)) {
    if (pos==1) {
      cat ("Who is significant with only", pos, "positive  sample  in this
library (p<=0.001):");
    } else {
      cat ("Who is significant with only", pos, "positives samples in this
library (p<=0.001):");
    }
    pass=0;
    for (i in 1:nrow(pMatrix)) {
      id=order (pMatrix[i,], decreasing=TRUE);
      if (pMatrix[i,id[pos]] <= 0.001) {
```

```
        cat ("\n", rownames(pMatrix)[i], "with a p-value of", pMatrix[i,
 id[pos]]);
        pass=pass+1;
      }
    }
    if (pass==0) {
      cat ("none !\n");
    } else {
      cat ("\n");
    }
    cat ("\n");
  }
}
```

Sample	27dupC	27insCCCC	26insG	28dupA	23delinsAT	WT	ALL	Ratio	
MUC-Sample01	2	0	0	0	0	4103	4105	0,04872107	
MUC-Sample02	1	0	0	0	0	3843	3845	0,0260078	
MUC-Sample03	0	0	0	0	0	1072	1073	0	
MUC-Sample04	1	10	0	0	0	3797	3814	0,02621919	
MUC-Sample05	1	0	0	0	0	3068	3069	0,0325839	
MUC-Sample06	3	0	0	0	0	3279	3282	0,09140768	
MUC-Sample07	38	0	0	0	0	2894	2933	1,29560177	p<=0.001
MUC-Sample08	0	0	0	0	0	1453	1453	0	
MUC-Sample09	50	0	0	0	0	2435	2485	2,01207243	p<=0.001
MUC-Sample10	0	0	17	0	0	1948	1948	0	
MUC-Sample11	37	0	0	0	0	2922	2960	1,25	p<=0.001
MUC-Sample12	59	0	0	0	0	5486	5547	1,063638	p<=0.001
MUC-Sample13	1	0	0	0	0	2266	2267	0,04411116	
MUC-Sample14	1	0	0	0	0	2184	2186	0,04574565	
MUC-Sample15	22	0	0	0	0	3330	3352	0,65632458	p<=0.001
MUC-Sample16	146	0	0	0	0	3141	3289	4,43903922	p<=0.001

Supplementary Table 1 – Example of the output of the bioinformatic run “Mutation Counter” Table with results of the Fischer statistic test comparing the number of occurrences of the mutation 27dupC for 16 samples. A p-value <0.001 (in red in the table) was considered as significative, meaning the sample contains a significantly higher number of reads presenting the variation compared to the other samples in the same run, and was considered as a positive carrier of the variation. This test was performed for each variant listed in the manuscript and in the first line of this table. MUC-Sample04 was positive for the mutation 27insCCC and MUC-Sample10 for the mutation 26insG.

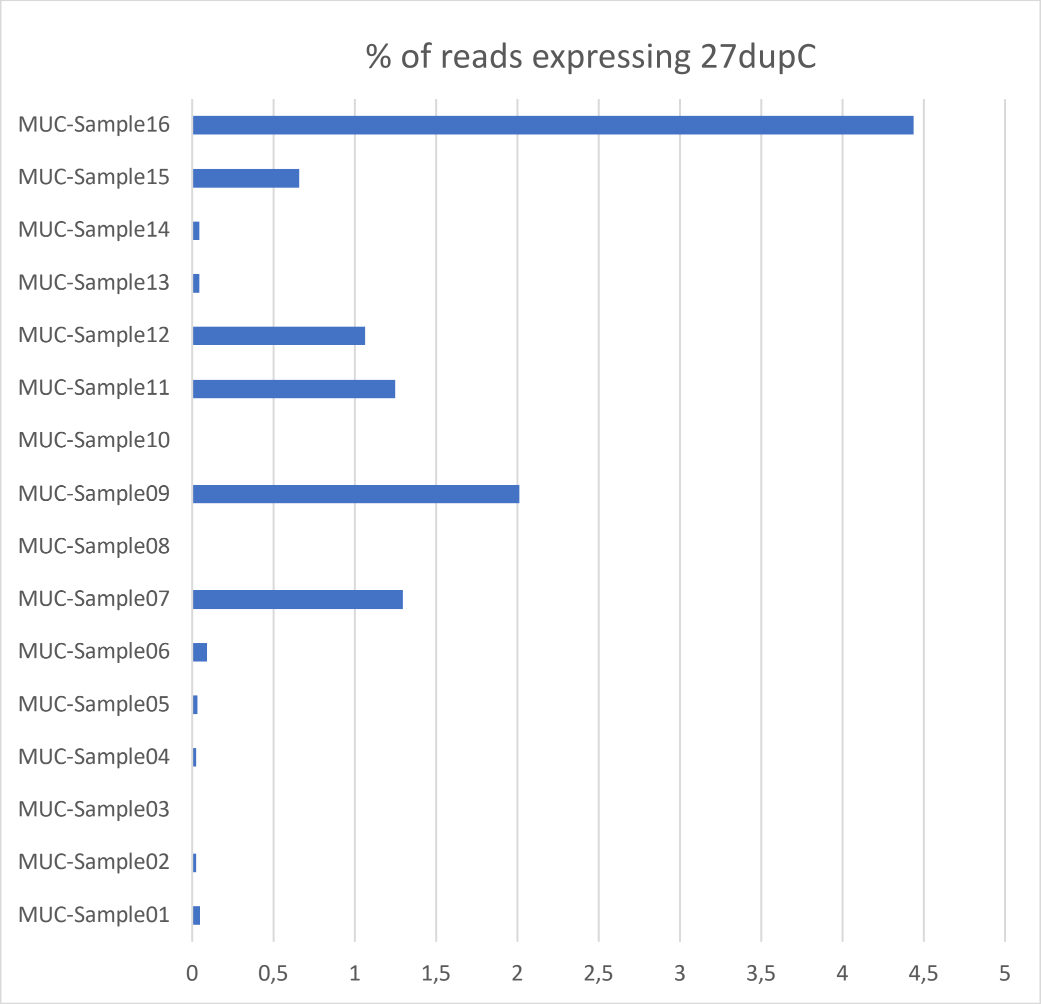

Supplementary Figure 1 – Graphical representation of the % of reads expressing the mutation 27dupC. MUC-Sample07, 09, 11, 12, 15 and 16 were positive carriers of the mutation (see Supplementary File S2)

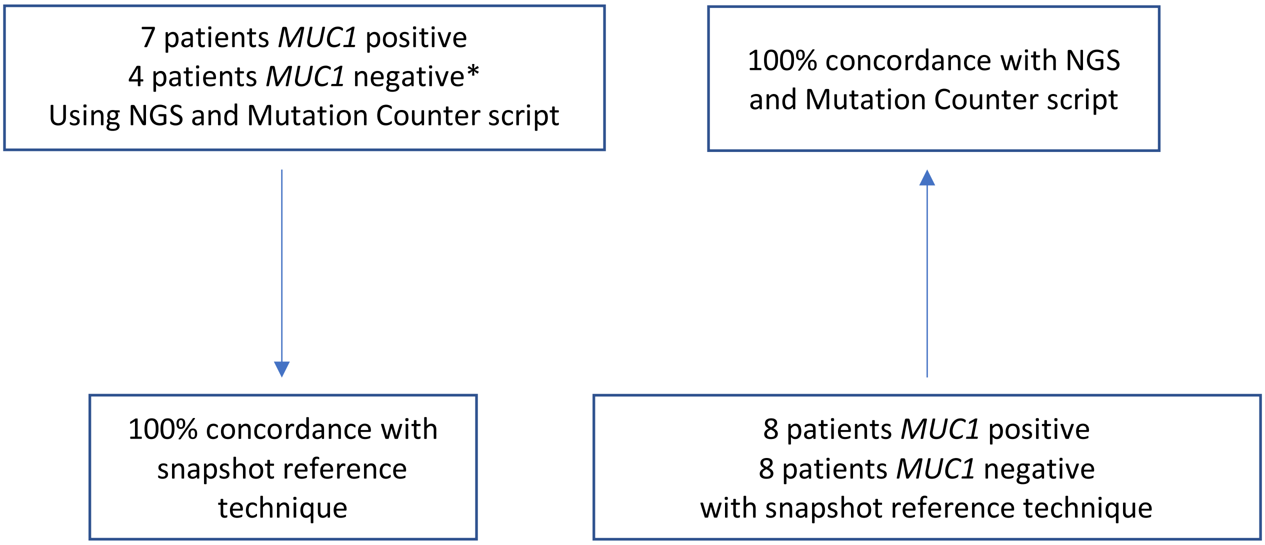

Supplementary Figure 2 - Validation of the Mutation Counter script

- 11 (7 positive/4 negative with familial history) samples analyzed in NGS were anonymously sent for confirmation with snapshot reference technique
- 16 (8 positive and 8 negative) samples confirmed with snapshot reference technique were anonymously analyzed using NGS and Mutation Counter script

NGS, Next Generation Sequencing

*asymptomatic adult childs of an ADTKD-*MUC1* patient

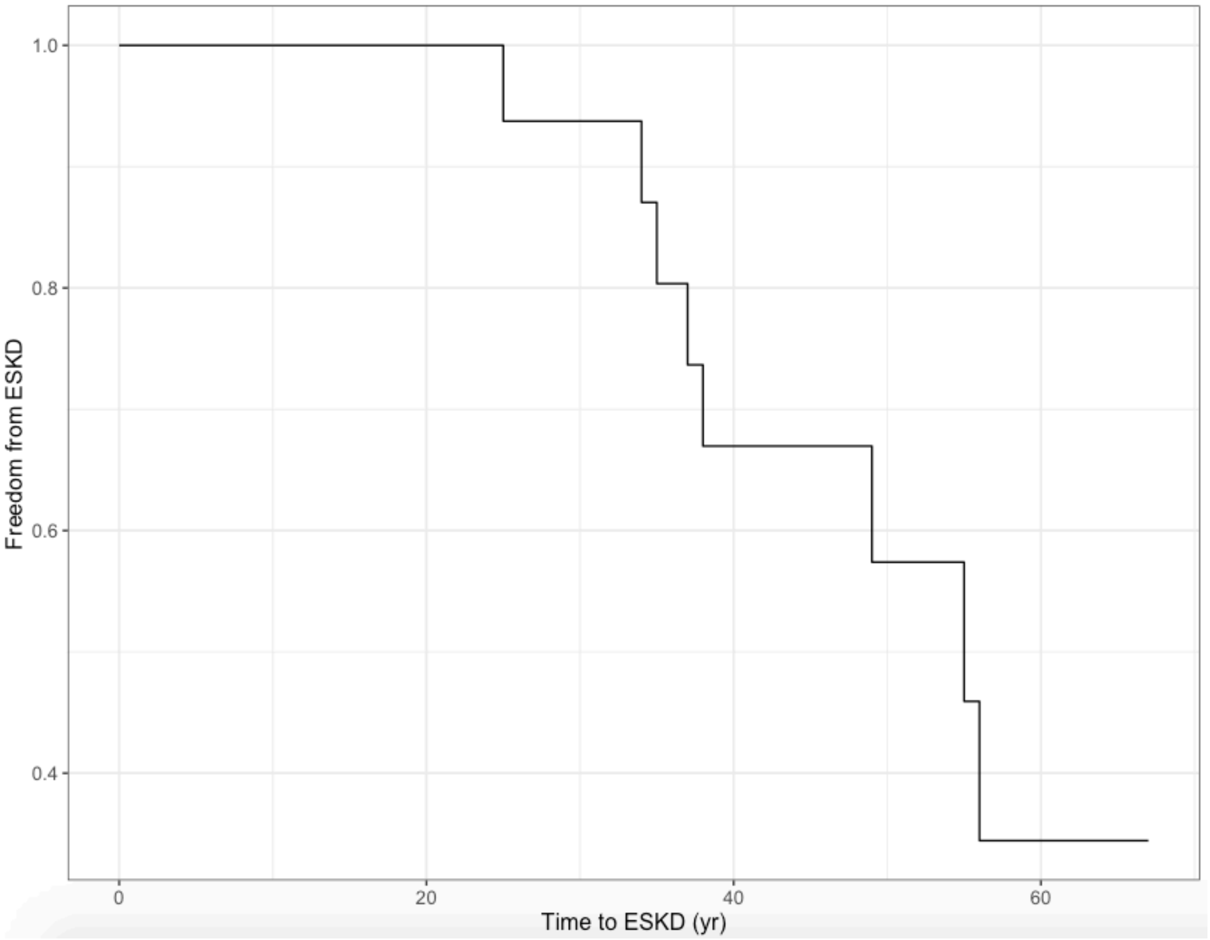

Supplementary Figure 3 - Freedom from end-stage kidney disease (ESKD). Kaplan Meier curve of renal survival in patients with ADTKD-MUC1. Median renal survival was 37.5 years[35;50.5]. Censored: ESKD has not occurred during the follow-up time.
